# In Vitro Evaluation of Anti-Inflammatory and Antioxidant Properties of a Novel Calcium Alginate–Zinc Hemostatic Biomaterial

**DOI:** 10.3390/jfb17050242

**Published:** 2026-05-11

**Authors:** Tanja Lunić, Marija Rakić, Maria Sbeih, Marina Samardzic, Céline des Courtils, Biljana Božić Nedeljković

**Affiliations:** 1 Institute of Physiology and Biochemistry “Ivan Djaja”, Faculty of Biology, University of Belgrade, 11000 Belgrade, Serbia; tanja.lunic@bio.bg.ac.rs (T.L.); marija.mandic@bio.bg.ac.rs (M.R.); 2 Laboratoires Brothier, 92000 Nanterre, France; maria.sbeih@brothier.com (M.S.); marina.samardzic@brothier.com (M.S.)

**Keywords:** hemostasis, calcium alginate, zinc ions, neurosurgery, neuroinflammation, microglia

## Abstract

Achieving hemostasis is crucial in neurosurgery, yet conventional methods are not always feasible, making topical hemostatic agents necessary. Current resorbable hemostatic agents allow effective hemostasis but must remain in situ to prevent rebleeding. This can provoke foreign body reactions leading to prolonged microglia-mediated neuroinflammation, which may exacerbate damage and delay recovery. It highlights the need for new hemostatic materials that can be removed after controlling bleeding while being safe for neurons and microglia. One candidate is Hemo-Ionic, a non-resorbable hemostatic compress composed of calcium alginate and zinc (Zn^2+^). Hemo-Ionic previously demonstrated effective in vitro and in vivo hemostasis, comparable to Surgicel and TachoSil, and pro-repair properties. In this study, Hemo-Ionic’s effect on neuronal and microglial cells was investigated in vitro. Results showed that Hemo-Ionic preserved cell viability and had an antioxidant capacity through protection from lipid peroxidation. Hemo-Ionic also reduced nitric oxide and pro-inflammatory cytokines (IL-6, IL-1β and TNF-α) expression and release by lipopolysaccharide (LPS)-stimulated microglial cells. Finally, neuronal viability was restored when exposed to supernatants of Hemo-Ionic-treated microglia. These findings indicate that Hemo-Ionic’s safety and capacity to reduce neuroinflammation, combined with its hemostatic efficacy and non-resorbable nature, make it a promising alternative to resorbable hemostatic agents used in neurosurgery.

## 1. Introduction

Effective hemostasis during neurosurgery is critical to maintain surgical visibility, minimize blood loss and lower the risk of postoperative intracranial hemorrhage, which can lead to increased intracranial pressure, seizures, or neurological deficits [1,2,3]. Conventional hemostasis methods include mechanical means (compression, clipping, sutures) or thermal means (electrocoagulation) [1,2] that are risky for critical neurovascular structures. Therefore, several topical hemostatic agents are used to achieve bleeding control in neurosurgery [1,4]. They include resorbable patches of oxidized regenerated cellulose (e.g., Surgicel™), gelatin matrices (e.g., Floseal^®^, Surgiflo^®^), collagen sponges (TachoSil^®^), fibrin-based sealants (e.g., Evicel^®^) or synthetic sealants (e.g., Duraseal^®^) [1,2,4,5]. The total USA market for hemostatic agents used in surgery is estimated to be between 8 and 9 billion dollars in 2024 [6,7]. All these agents are composed of resorbable materials and achieve hemostasis through mechanical action, by sealing the breach and supporting platelet aggregation. Some of them additionally exert biological hemostatic action by promoting fibrin clot formation [1,2]. They cannot be removed from the bleeding site as their removal disturbs the blood clot and causes rebleeding [5]. Left in situ after closing the surgical site, they can cause foreign body reactions (formation of granuloma or pseudotumor, giant cell infiltration, abscess-like reaction, fibrosis, etc.) [1,2,5,8,9]. These resorbable hemostatic agents can also cause prolonged neuroinflammation, i.e., sustained microglial release of mediators, such as reactive oxygen species (ROS), nitric oxide (NO) and pro-inflammatory cytokines, which leads to oxidative stress and exacerbates neuronal damage [1,2,5,8,9,10,11]. Numerous adverse events of resorbable hemostatic agents were reported in the literature: foreign body reactions, compression on neural structures, allergic reactions, etc. [5,8,10,12,13,14,15,16,17].

This highlights the need for new hemostatic agents that are safe for neuronal and microglial cells and can be removed before closing the surgical site, thus avoiding foreign body reactions. Calcium (Ca^2+^) alginate compress, derived from brown seaweed, is widely used in different surgeries, except in neurosurgery. This is probably because its safety was never evaluated in contact with microglia and neurons.

One potential candidate for neurosurgery is Hemo-Ionic, a non-resorbable hemostatic compress under development, made of calcium alginate and zinc (Zn^2+^). Compared to Surgicel and TachoSil, Hemo-Ionic demonstrated equivalent hemostasis through in vitro whole-blood coagulation assay and pig liver lesion studies. It also promoted better wound healing in mice [18]. Hemo-Ionic’s hemostatic efficacy relies on mechanical action, by supporting platelet aggregation, and on biological action thanks to its release of Ca^2+^ and Zn^2+^ ions. Ca^2+^ ions act as potent platelet and cell activators and as a key coagulation factor (factor IV) [19,20,21,22]. Zn^2+^ ions further stimulate calcium signaling [23,24,25] and play a direct role in platelet aggregation and coagulation cascades [21,26,27]. After releasing Ca^2+^ and Zn^2+^, Hemo-Ionic can be safely removed from the surgical site within ten minutes. Thanks to its gelation upon contact with blood, Hemo-Ionic detaches from the blood clot without damaging it, thereby preventing rebleeding [18]. Beyond hemostasis, both Ca^2+^ and Zn^2+^ ions may also support axonal regeneration [28,29] and regulate neural stem cell differentiation [29,30], while Zn^2+^ ions further modulate inflammation and oxidative stress in the brain [29,31].

As the hemostatic efficacy of Hemo-Ionic was already demonstrated [18], the aim of this study was to investigate Hemo-Ionic’s biocompatibility and its effect on neuronal and microglial cells in vitro, as it may be a promising alternative to current hemostatic resorbable agents in neurosurgery.

## 2. Materials and Methods

### 2.1. Description of the Dressing

Hemo-Ionic was first described in Ponsen et al. [18]. It is a non-woven calcium alginate compress enriched with zinc gluconate by spraying. Alginates are polysaccharides composed of two monomers of L-guluronic acid (G) and D-mannuronic acid (M). The ratio of these two monomers determines the physicochemical properties of the alginate. The predominantly guluronic calcium alginate fibers that make up Hemo-Ionic are obtained after extrusion of a solution of sodium alginate/cupric chlorophyllin (E141) through a die in a calcium chloride bath. The fibers produced are carded and then needle-punched to obtain a calcium alginate compress. The compress is then enriched with zinc gluconate by spraying. Hemo-Ionic is a product in development at Laboratoires Brothier (Nanterre, France). Table 1 presents the general composition, structure, and physicochemical properties of Hemo-Ionic as well as its Ca^2+^ and Zn^2+^ ion release. Fiber morphology was evaluated using scanning electron microscopy (SEM), while diameters were calculated using inverted phase-contrast microscopy images at 400× magnification and NIS-Elements software 5.11.x. Absorption capacity was quantified according to standard ISO 13726-1:2002 [32], and tensile strength and elongation were quantified according to standard ISO 29073-3:1992 [33]. Calcium alginate content was determined through High-Performance Liquid Chromatography (HPLC) and its M/G ratio through Nuclear Magnetic Resonance (NMR). Ca^2+^ and Zn^2+^ release were measured by inductively coupled plasma optical emission spectroscopy (ICP-OES), following standard ISO 11885:2007 [34]. Heavy metals and trace elements were determined using Atomic Absorption Spectroscopy (AAS).

### 2.2. Dressing Extract Preparation

For extract preparation, Hemo-Ionic was cut into 1 cm^2^ pieces and incubated either in PBS (for antioxidant assays) or in complete RPMI 1640 medium (for cell culture assays), using the same material-to-liquid ratio of 3 cm^2^/mL. Accordingly, each 1 cm^2^ piece was immersed in approximately 330 μL of the respective extraction medium. Extraction was carried out for 24 h at 37 °C. After incubation, the extracts were collected and filtered through a 0.22 μm syringe filter to remove any residual fibers and were used immediately in subsequent assays. This procedure was done in accordance with the ISO 10993-12:2021 [35] standard for the evaluation of medical devices and aims to obtain the releasable fraction of the dressing under defined in vitro conditions.

### 2.3. Antioxidant Assays

#### 2.3.1. β-Carotene Bleaching Assay

Briefly, an emulsion containing linoleic acid (6.25 μL), Tween 40 (50 mg), and β-carotene in chloroform (125 μL, 4 mg/mL) was prepared. After complete mixing, chloroform was evaporated using a rotary evaporator at 40 °C, and the residue was reconstituted as previously described [36]. Then, 200 μL of the emulsion was mixed with 28 μL of dressing extract, standards (butylated hydroxyanisole, BHA; butylated hydroxytoluene, BHT; and vitamin C) dissolved in PBS at a working concentration of 500 μg/mL, or PBS (negative control).

Absorbance was measured at 490 nm immediately (t0) and after 120 min of incubation (t120) at room temperature, using a Multiskan Sky microtiter plate reader (Thermo Scientific, Vantaa, Finland). Inhibition of β-carotene bleaching by each sample was calculated using the following equation:

Inhibition (%) = [(As120 − Ac120)/(Ac0 − Ac120)] × 100, where As120 and Ac120 are absorbances of the sample and negative control at t120, respectively, and Ac0 is the absorbance of the negative control at t0.

#### 2.3.2. ABTS Assay

ABTS assay was adapted from Miller et al. [37]. Briefly, a solution of ABTS radicals (7 mM) was prepared 12–16 h prior to the experiment. 200 μL of ABTS solution was mixed with 5 μL of dressing extract, standards (BHA, BHT and vitamin C dissolved in PBS), or distilled water (negative control) and incubated for 30 min at 30 °C. Absorbance was measured at 734 nm using a Multiskan Sky microtiter plate reader (Thermo Scientific, Vantaa, Finland). Inhibition of ABTS radicals by each sample was calculated using the following equation:

Inhibition (%) = (Ac − As)/Ac × 100, where Ac is the absorbance of the negative control and As is the absorbance of the sample.

#### 2.3.3. DPPH Assay

DPPH assay was adapted from Blois et al. [38]. Briefly, a solution of methanolic DPPH solution (40 μg/mL) was freshly prepared. 180 μL of DPPH solution was mixed with 20 μL of dressing extract, standards (BHA, BHT and vitamin C dissolved in PBS), or PBS (negative control). Absorbance was measured at 517 nm after a 30 min incubation using a Multiskan Sky microtiter plate reader (Thermo Scientific, Vantaa, Finland). Inhibition of DPPH radicals by each sample was calculated using the following equation:

Inhibition (%) = (Ac − As)/Ac × 100, where Ac is the absorbance of the negative control and As is the absorbance of the sample.

#### 2.3.4. Total Reducing Power Assay

Total Reducing Power (TRP) assay was adapted from Oyaizu et al. [39]. Briefly, 40 μL of phosphate buffer (0.2 M, pH 6.6) combined with 40 μL of 1% potassium ferricyanide (III) was mixed with 20 μL of dressing extract, standards (BHA, BHT and vitamin C dissolved in PBS), or PBS (negative control). After a 20 min incubation at 45 °C, 40 μL of trichloroacetic acid (10%, *w*/*v*), 40 μL of distilled water, and 8 μL of 0.1% iron (III) chloride were added, followed by a 10 min incubation at room temperature. Absorbance was measured at 700 nm using a Multiskan Sky microtiter plate reader (Thermo Scientific, Vantaa, Finland). Results were normalized against vitamin C (ascorbic acid) values, and Total Reducing Power was expressed as μmol of Ascorbic Acid Equivalents (AAE) per gram of dry extract (μmol AAE/g dry extract).

### 2.4. In Vitro Assays

#### 2.4.1. Cell Culture

All cells were obtained from American Tissue Culture Collection (ATCC, Manassas, VA, USA). BV2 (murine microglia) and SH-SY5Y (human neuroblastoma) cell lines were maintained in RPMI-1640 medium supplemented with 10% fetal bovine serum (FBS), 1% glucose, and 1% penicillin-streptomycin. The cells were incubated at 37 °C in a humidified atmosphere with 5% CO_2_.

#### 2.4.2. Cell Treatment with Dressing Extracts

Mouse microglial (BV2) and human neuronal (SH-SY5Y) cells were seeded in 96-well plates at a density of 1 × 10^4^ cells/well in 100 μL complete medium for MTT/Griess assays, and in 6-well plates at a density of 1 × 10^6^ cells/well for qRT-PCR analysis. Cells were incubated for 24 h to allow attachment, after which the culture medium was replaced with 200 μL/well of Hemo-Ionic dressing extract or complete medium (Control) and treated for 24 h (SH-SY5Y) or 48 h (BV2). For experiments on LPS-stimulated microglia, BV2 cells were treated with LPS (final concentration of 10 μg/mL) alone or simultaneously with Hemo-Ionic dressing extract for 48 h. After this treatment period, the medium was replaced with fresh culture medium without LPS or extract, and BV2 cells were further incubated for an additional 48 h before performing downstream assays. Non-stimulated cells cultured in complete medium served as negative controls.

The LPS concentration was determined in preliminary experiments by measuring nitric oxide production in response to concentrations ranging from 0.1 to 10 μg/mL, with 10 μg/mL eliciting the highest response.

#### 2.4.3. MTT Assay

The metabolic activity of SH-SY5Y and BV2 cells was determined using an MTT assay [40]. Briefly, 10 μL of sterile MTT solution (5 mg/mL) was added to 100 μL of culture medium in each well, resulting in a final MTT concentration of 0.5 mg/mL. Cells were incubated for 3 h at 37 °C, after which 100 μL of 10% SDS containing 1 N hydrochloric acid was added to dissolve the formazan crystals. Absorbance was measured at 540 nm using an Agilent BioTek Epoch microplate reader (Agilent Technologies Inc., Santa Clara, CA, USA). Results were expressed as a percentage of the control group, normalized to 100%.

#### 2.4.4. NO Production Assay

Griess assay [41] was performed by mixing 50 μL of cell supernatants with 50 μL of Griess reagent. After 10 min of incubation, absorbance was measured at 540 nm using a Microplate Reader Agilent BioTek Epoch microplate reader (Agilent Technologies Inc., Santa Clara, CA, USA). Results are presented as nitrite concentration (μM) equivalent to NO production.

#### 2.4.5. Quantification of Cytokine Levels

IL-1β (ab197742; Abcam, Cambridge, UK), IL-6 (ab222503; Abcam, Cambridge, UK) and TNF-α (ab208348; Abcam, Cambridge, UK) cytokine levels were quantified in cell supernatants using enzyme-linked immunosorbent assay (ELISA) kits according to the manufacturer’s instructions. Results are reported in pg/mL.

#### 2.4.6. RT-qPCR

Relative mRNA expression levels of TNF-α, iNOS, COX-2, and IL-1β were quantified by quantitative reverse transcription-polymerase chain reaction (qRT-PCR). Briefly, total RNA was extracted from samples using the Monarch^®^ Total RNA Miniprep Kit (New England Biolabs, Ipswich, MA, USA) according to the manufacturer’s instructions. RNA concentration and purity were determined using a NanoDrop spectrophotometer (Agilent Technologies Inc., Santa Clara, CA, USA). Complementary DNA (cDNA) was synthesized from the isolated RNA using the High-Capacity cDNA Reverse Transcription Kit (Thermo Fisher Scientific, Waltham, MA, USA) according to the manufacturer’s instructions. qRT-PCR was performed using PowerUp™ SYBR™ Green Master Mix (Thermo Fisher Scientific, Waltham, MA, USA) and primers listed in Table 2. Amplification was performed on a StepOnePlus™ Real-Time PCR System. Gene expression values were normalized to Glyceraldehyde-3-phosphate dehydrogenase (GAPDH) expression in the same samples.

#### 2.4.7. Microglial Supernatant Transfer Model

Supernatants from LPS-stimulated BV2 cells treated with dressing extract were transferred to a 96-well microplate seeded with SH-SY5Y cells (1 × 10^4^ cells per well). Following a 24 h incubation period, neuronal metabolic activity was assessed using the MTT assay, as previously described.

### 2.5. Statistical Analysis

Statistical analysis was performed using GraphPad Prism v11. Data shown are one representative experiment from three independent experiments with at least *n* = 3. Normality was tested using the Shapiro–Wilk test and comparisons between groups were made using an unpaired Student’s *t*-test. A *p*-value of less than 0.05 was considered statistically significant.

## 3. Results

### 3.1. Antioxidant Activity of Hemo-Ionic

ABTS, DPPH and TRP assays revealed weak antioxidative activity of Hemo-Ionic compared to the three standards BHT (butylated hydroxytoluene), BHA (butylated hydroxyanisole) and Vitamin C (Table 3). Conversely, Hemo-Ionic strongly inhibited β-carotene bleaching by 61%, demonstrating efficient antioxidant activity.

### 3.2. Effect of Hemo-Ionic on Cell Viability

Viability of neurons SH-SY5Y and microglia BV2 cells was not altered by Hemo-Ionic extract, with 92.7% and 96.2% of metabolic activity, respectively, compared to Control (Table 4). This indicates that Hemo-Ionic is non-cytotoxic on neural cells.

### 3.3. Effect of Hemo-Ionic on Microglia-Driven Inflammation

**Figure 1:**
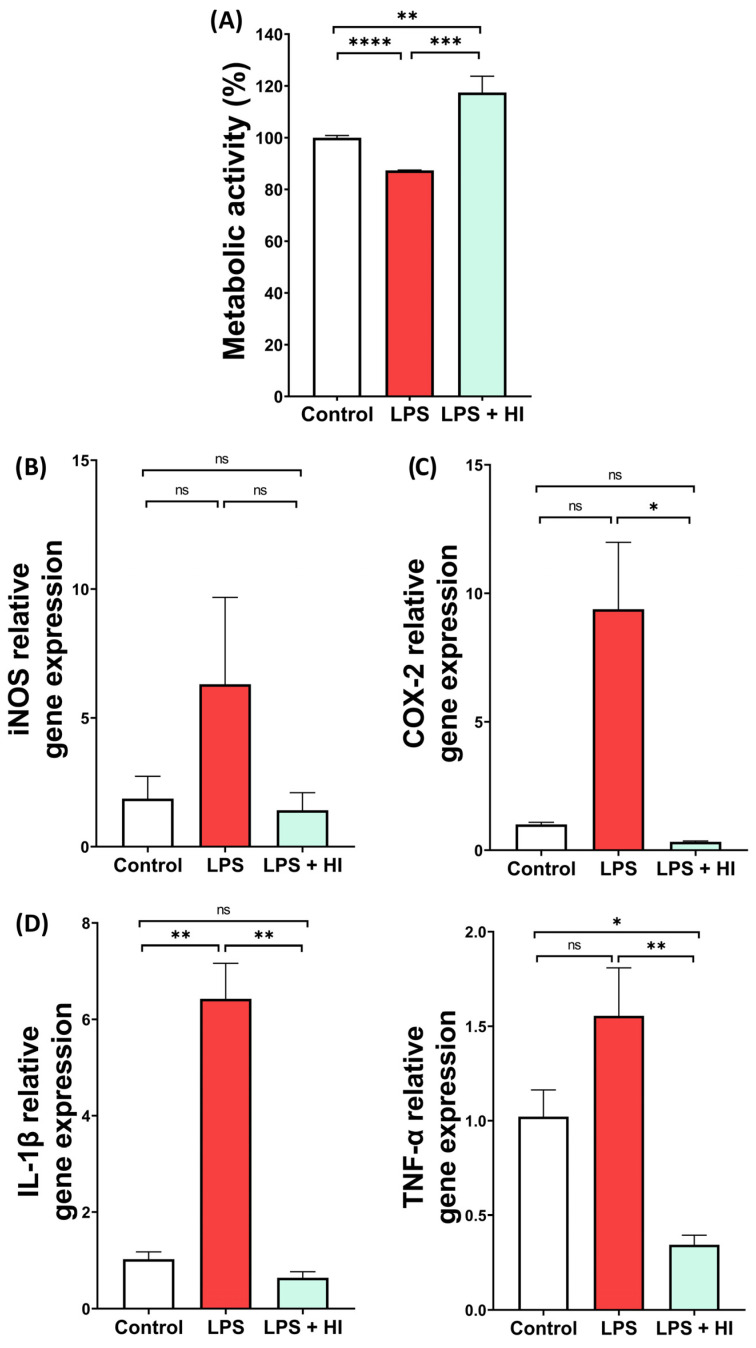
Effect of Hemo-Ionic on microglial metabolic activity and gene expression. Cell metabolic activity assay (**A**) and relative gene expression of (**B**) iNOS, (**C**) COX-2 and (**D**) IL-1β and TNF-α in unstimulated (Control), LPS-stimulated (LPS) and LPS-stimulated Hemo-Ionic-treated (LPS + HI) BV2 cells. Data are presented as mean ± SEM (n = 3). Statistical significance is indicated above the connected groups. ns = not significant, * *p* < 0.05, ** *p* < 0.01, *** *p* < 0.001, **** *p* < 0.0001.

Following LPS stimulation, the metabolic activity of microglia BV2 cells was markedly reduced compared to Control non-stimulated cells. Adding Hemo-Ionic extract restored their viability in a significant manner (Figure 1A). As LPS activates microglia into a pro-inflammatory state, pro-inflammatory gene expression of BV2 cells was quantified by RT-qPCR. As expected, inducible nitric oxide synthase (iNOS), responsible for producing nitric oxide (NO) under inflammatory stimuli, was increased in BV2 cells after LPS stimulation. Hemo-Ionic extract decreased this expression to control levels, although not significantly (Figure 1B). Expression of the inducible enzyme cyclooxygenase-2 (COX-2), which generates pro-inflammatory prostaglandins, was overexpressed by LPS-activated BV2 cells and strongly reduced with Hemo-Ionic extract (Figure 1C). Similarly, pro-inflammatory cytokines Interleukin (IL)-1β and Tumor Necrosis Factor (TNF)-α were overexpressed after LPS stimulation and significantly downregulated by Hemo-Ionic extract (Figure 1D).

**Figure 2:**
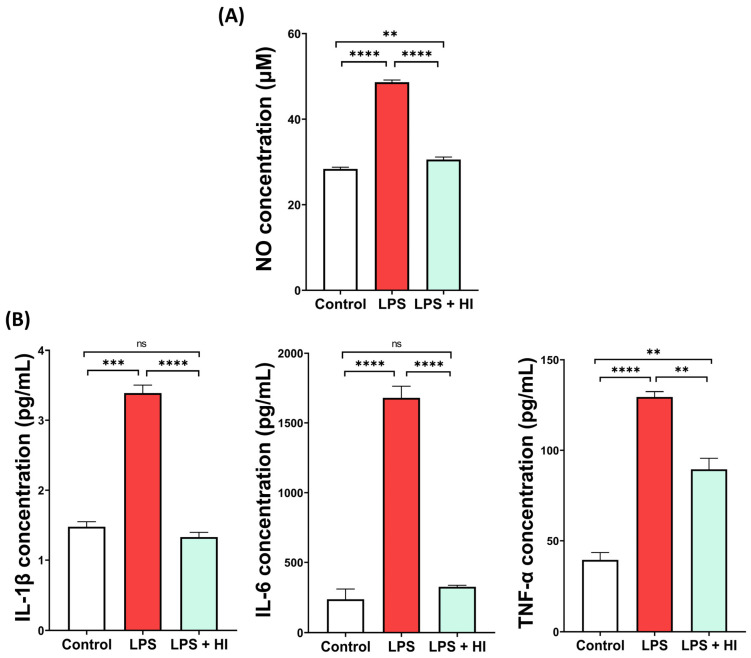
Effect of Hemo-Ionic on release of pro-inflammatory mediators by microglia. Quantification of (**A**) NO release and (**B**) IL-1β, IL-6 and TNF-α secretion, in unstimulated (Control), LPS-stimulated (LPS) and LPS-stimulated Hemo-Ionic-treated (LPS + HI) BV2 cells. Data are presented as mean ± SEM (n = 3–4). Statistical significance is indicated above the connected groups. ns = not significant, ** *p* < 0.01, *** *p* < 0.001, **** *p* < 0.0001.

To support these results, secretion of pro-inflammatory factors was also quantified. As expected, LPS stimulation significantly increased secretion of NO (Figure 2A) and cytokines IL-1β, IL-6 and TNF-α by BV2 cells (Figure 2B). Treatment with Hemo-Ionic extract significantly reduced the production of these mediators (Figure 2A,B), even restoring levels comparable to Control for NO, IL-1β and IL-6.

Taken together, these results show that Hemo-Ionic restores microglial viability after LPS activation and acts by reducing pro-inflammatory mediators at both transcriptional and translational levels.

### 3.4. Effect of Hemo-Ionic on Neuronal Cells

**Figure 3:**
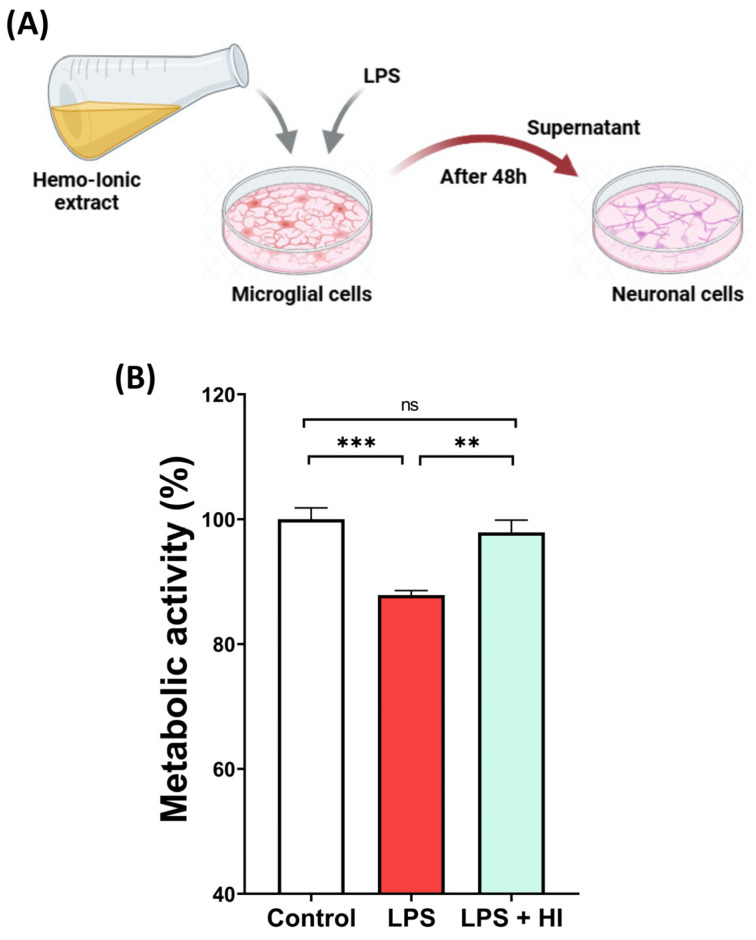
Effect of conditioned media from Hemo-Ionic-treated microglia on neuronal metabolic activity. (**A**) Supernatants of microglial BV2 cells were transferred to SH-SYS5Y neuronal cells. Created with BioRender.com (**B**) Effect of supernatants from unstimulated (Control), LPS-stimulated (LPS) and LPS-stimulated Hemo-Ionic-treated (LPS + HI) BV2 cells on the metabolic activity of SH-SY5Y neurons. Data are presented as mean ± SEM (n = 3). Statistical significance is indicated above the connected groups. ns = not significant, ** *p* < 0.01, *** *p* < 0.001.

To evaluate how downregulating microglia inflammation affects neuronal cells, supernatants from LPS-stimulated BV2 microglia, with or without Hemo-Ionic treatment, were transferred to SH-SY5Y neuron cultures (Figure 3A). Neurons exposed to supernatants from LPS-stimulated BV2 cells showed a significant decrease in viability compared to those exposed to supernatants from unstimulated BV2 cells (Control). In contrast, neurons exposed to supernatants from LPS-stimulated BV2 cells treated with Hemo-Ionic showed a significant recovery in viability, restoring it to control levels (Figure 3B). These findings demonstrate the neuroprotective potential of Hemo-Ionic.

## 4. Discussion

Effective hemostasis is critical in neurosurgery and often cannot rely on conventional methods, making local hemostatic agents necessary [1,4]. Hemostatic agents, composed of resorbable materials, must remain in place as they provide support for platelet aggregation, and their removal disturbs the blood clot and causes rebleeding [5]. Left in situ, they can cause foreign body reactions leading to prolonged neuroinflammation driven by microglia, which leads to oxidative stress and exacerbates neuronal damage [1,2,5,8,9,10,11]. Consequently, there is a need for a hemostatic agent that would be safe for neuronal and microglial cells, and that can be removed from the bleeding site.

Hemo-Ionic is a newly developed non-resorbable calcium alginate compress enriched with Zn^2+^ ions. We have demonstrated its strong hemostatic efficacy, which persisted after its removal from the bleeding site [18]. This study aimed to evaluate Hemo-Ionic’s biocompatibility and its effect on neuronal and microglial cells in vitro, as it may be a promising alternative to current hemostatic resorbable agents in neurosurgery.

We first investigated the antioxidant activity of Hemo-Ionic extract, as neuronal membranes are markedly vulnerable to ROS-induced damage due to their high content in polyunsaturated fatty acids with carbon double bonds, which serve as target sites for ROS [42,43]. Hemo-Ionic exhibited a high antioxidant activity in the β-carotene bleaching assay, which measures the ability to stabilize radicals involved in lipid peroxidation of cell membranes. This is particularly relevant for neurosurgery as lipid peroxidation increases following brain surgery and is considered a marker of secondary brain injury [44]. In contrast, the three other assays, which measure direct ROS neutralization (ABTS and DPPH) or reduction mechanisms (TRP), did not show significant antioxidant activity. This suggests that Hemo-Ionic extract may be more effective at limiting lipid peroxidation-related processes than at direct radical scavenging or reducing reactions under the tested conditions.

The indirect antioxidant activity of Hemo-Ionic could either be attributed to calcium alginate, which inhibits lipid peroxidation [45] and/or to Zn^2+^ ions, which have been reported to stabilize cell membranes, modulate Nrf2 signaling, a key regulator of the cellular antioxidant response [31] and reduce lipid peroxidation in brains of rats and mice [29,46,47]. However, since the involvement of Zn^2+^ ions was not directly investigated using chelating approaches (such as EDTA), mechanisms underlying the antioxidant effects of the Hemo-Ionic extract remain to be investigated in future studies.

The impact of released ions on neural cell viability should also be considered, as calcium, although essential for normal neuronal function, may contribute to excitotoxicity when dysregulated. Excess extracellular Ca^2+^ and increased influx, particularly via glutamate receptor overactivation, can lead to intracellular calcium overload and neuronal cell death [48]. Similarly, excessive Zn^2+^ ions also contribute to several neurotoxic pathways [29]. Hemo-Ionic extract, containing Ca^2+^ and Zn^2+^ ions released by the dressing (Table 1), was non-cytotoxic to neuronal cells (SH-SY5Y) and microglia (BV2), including non-activated and LPS-activated microglia, confirming its biocompatibility with neural tissue. It even improved the metabolic activity of LPS-activated microglia, suggesting a protective effect under inflammatory conditions. These findings align with a prior cytotoxicity assay conducted on endothelial colony-forming cells in which Hemo-Ionic preserved cell metabolic activity, contrary to Surgicel and TachoSil [18]. Other studies demonstrated that calcium alginate was non-cytotoxic to primary fibroblasts [49] and monocytes [50]. Nevertheless, some limitations of the present study should be considered. The biological evaluation was performed in two cell lines, BV2 microglia and SH-SY5Y neuroblastoma cells, which do not fully reflect the complexity of the neural tissue environment in vivo. In addition, the neuronal model used here consisted of undifferentiated SH-SY5Y cells, which represent a simplified in vitro model and may be less responsive to excitotoxic stimuli than differentiated neuronal cells or primary neurons [51]. Therefore, although these models are well established and widely used for initial in vitro screening, future studies should include more physiologically relevant neuronal and glial models.

Concerning the Hemo-Ionic effect on LPS-activated microglia, microglial release of pro-inflammatory mediators NO, IL-6, IL-1β and TNF-α was significantly reduced. This effect was also corroborated at the transcriptional level: expression of iNOS (ns), IL-1β and TNF-α, along with COX-2, was significantly downregulated. This aligns with findings from Hongxia et al., who reported that zinc ions significantly reduced iNOS, TNF-α and COX2 expression in LPS-treated BV2 cells [52]. A possible explanation may involve mechanisms previously associated with zinc, such as the upregulation of zinc-finger protein A20, which has been reported to negatively regulate NF-κB signaling and thereby reduce the expression of pro-inflammatory mediators in microglia [52,53]. However, this mechanism was not directly investigated in the present study. Interestingly, in our study, IL-6 expression remained unchanged despite a reduction in its cytokine secretion, suggesting that Hemo-Ionic may affect IL-6 via a post-transcriptional mechanism.

Apart from Zn^2+^, calcium alginate may also contribute to Hemo-Ionic’s anti-inflammatory effect. Contradictory findings have been reported: pure calcium alginate extract exhibited a pro-inflammatory effect on human macrophages [50] while its hydrogel formulation promoted macrophage polarization towards the M2 anti-inflammatory phenotype and decreased TNF-α production in LPS-activated monocytes [54]. Further studies should be conducted to discriminate between calcium alginate and Zn^2+^ ions contribution to Hemo-Ionic’s anti-inflammatory effect.

Finally, we assessed whether Hemo-Ionic could protect neurons from microglial inflammation. Previous studies showed that neuronal damage occurs upon chronic or excessive microglial activation [11] and that TNF-α further promotes neuronal phagocytosis by microglia, followed by neuronal loss [55]. Our results show that neuronal metabolic activity significantly improved when in contact with Hemo-Ionic-treated microglia, likely due to the decreased secretion of pro-inflammatory mediators. These results suggest that Hemo-Ionic could have a neuroprotective effect in a clinical setting.

Apart from this protective effect of neurons against microglia-driven inflammation, the release of Ca^2+^ and Zn^2+^ ions by Hemo-Ionic may have a direct beneficial role on neurons, as they were shown to support axonal regeneration [28,29] and neural stem cell differentiation [29,30]. In more physiological contexts, the Hemo-Ionic release of Zn^2+^ ions may also help restore zinc levels, which are often depleted after CNS injury and are associated with poor functional outcomes, particularly in spinal cord injury [53].

A limitation of the study is the use of dressing extracts in contact with cells. This was necessary to avoid hypoxic effects from direct dressing contact with cell cultures. The use of dressing extracts is recommended by the ISO 10993-12:2021 [35] standard for medical device evaluation. Furthermore, treatment periods represent a standardized in vitro worst-case condition and do not fully replicate the temporary clinical application of the material in neurosurgery. However, concerning ion release by Hemo-Ionic, previous characterization [18] shows that the majority of Zn^2+^ and Ca^2+^ release occurs rapidly within the first minutes of contact, which is consistent with the expected intraoperative use window. Future studies are warranted to further validate the performance and safety of Hemo-Ionic compress material in clinically relevant neurosurgical settings. Another limitation would be the absence of mechanistic investigations allowing us to link anti-inflammatory and antioxidant properties of Hemo-Ionic’s extract to specific pathways. These investigations should be addressed in the future, along with the inclusion of a pure calcium alginate comparator to decipher the respective contributions of calcium alginate and Zn^2+^ ions.

## 5. Conclusions

Our in vitro data demonstrate that Hemo-Ionic extract is non-cytotoxic to neuronal and microglial cells and exerts antioxidant effects, particularly in terms of lipid peroxidation. It also reduces the inflammatory response of LPS-stimulated microglia. Conditioned media from Hemo-Ionic-treated microglia restored neuronal metabolic activity, suggesting indirect modulation under inflammatory conditions and a potential neuroprotective effect in more physiological settings.

Hemo-Ionic has a hemostatic efficacy comparable to Surgicel and TachoSil [18]. Unlike these resorbable hemostatics, Hemo-Ionic is removed upon achieving hemostasis, thanks to its release of Ca^2+^ and Zn^2+^, well-known clotting players [19,20,21,26,27]. This avoids the risks of foreign body reactions. Additionally, a 10 min Hemo-Ionic application on murine skin lesions promoted long-term tissue repair versus TachoSil and Surgicel [18].

Taken together, these properties position Hemo-Ionic as a promising candidate for neurosurgical hemostasis and justify further evaluation.

## Figures and Tables

**Table 1:** Main physicochemical characteristics of Hemo-Ionic. Pictures are Scanning Electron Microscopy images. Ca^2+^ and Zn^2+^ ion release are represented as concentration means, measured in culture medium at 10 min, 24 h and 48 h after adding Hemo-Ionic. Scale bar: 500 μm (left) and 20 μm (right). * Mean ± SD; ° Measured in NaCl 0.9%. HPLC: High-Performance Liquid Chromatography; NMR: Nuclear Magnetic Resonance; M: D-mannuronic acid; G: L-guluronic acid; ICP-OES: Inductively Coupled Plasma Optical Emission Spectroscopy; AAS: Atomic Absorption Spectroscopy.

Hemo-Ionic	
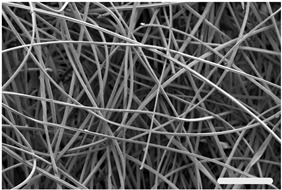	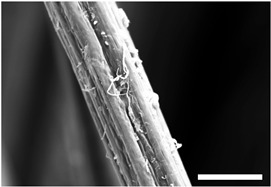
Composition	Non-absorbable calcium alginate fibers enriched with zinc ions
Format	10 × 20 cm
Structure	Needled non-woven compress
Sterilization	β-irradiation
Fiber shape	Multilobed
Fiber diameter *	28.8 μm ± 4.0
Fiber swelling (ratio dry/wet°)	4.05
Fluid absorption capacity *°	10.28 g/g ± 0.396
Tensile strength *°	6.65 N ± 0.59
Elongation at break *°	39.89 mm ± 3.09
HPLC	
Calcium alginate content	99.5%
NMR	
Alginate M/G ratio	0.46
ICP-OES	
Ion release at 10 min	[Ca^2+^]: 3.74 mM; [Zn^2+^]: 27.13 μM
Ion release at 24 h	[Ca^2+^]: 4.69 mM; [Zn^2+^]: 27.62 μM
Ion release at 48 h	[Ca^2+^]: 4.9 mM; [Zn^2+^]: 36.74 μM
AAS	
Heavy metal analysis	≤20 ppm
Arsenic analysis	≤1 ppm
Cadmium analysis	≤1.1 ppm
Iron content	≤250 ppm
Sodium content	<0.02%

**Table 2:** Primer sequences. F = Forward, R = Reverse.

Gene	Primers
*iNOS*	F: 5′-GCAGCCTGTGAGACCTTTG-3′ R: 5′-GCATTGGAAGTGAAGCGTTTC-3′
*COX-2*	F: 5′-CCACTTCAAGGGAGTCTGGA-3′ R: 5′-AGTCATCTGCTACGGGAGGA-3′
*IL-1β*	F: 5′-GCAACTGTTCCTGAACTCAACT-3′ R: 5′-ATCTTTTGGGGTCCGTCAACT-3′
*TNF-α*	F: 5′-TGGCCTCCCTCTCATCAGTT-3′ R: 5′-GCTTGTCACTCGAATTTTGAGAAG-3′
*GAPDH*	F: 5′-TTCACCACCATGGAGAAGGC-3′ R: 5′-GGCATGGACTGTGGTCATGA-3′

**Table 3:** Antioxidant activity of Hemo-Ionic assessed with four different assays. Data are presented as mean ± S.E.M. BHT: butylated hydroxytoluene; BHA: butylated hydroxyanisole.

	Antioxidant Assay			
Sample	ABTS Radical Inhibition (%)	DPPH Radical Inhibition (%)	TRP (μmoL AAE/g of Dry Extract)	β-Carotene Bleaching Inhibition (%)
BHT	100 ± 0.92	85.24 ± 0.94	130.85 ± 0.69	80.84 ± 0.39
BHA	100 ± 0.27	84.01 ± 0.37	118.43 ± 0.49	87.42 ± 0.18
Vitamin C	100 ± 00.45	87.86 ± 0.73	129.42 ± 0.41	14.19 ± 0.16
Hemo-Ionic	23.07 ± 0.72	6.89 ± 0.94	7.48 ± 0.10	61.17 ± 0.21

**Table 4:** Effect of Hemo-Ionic on cell metabolic activity. Cytotoxicity testing of culture medium alone (Control) or Hemo-Ionic extract (Hemo-Ionic) on the SH-SY5Y neuron cell line and BV2 microglia cell line, by MTT assay. Cells were exposed to Hemo-Ionic extract for 24 h (SH-SY5Y) or 48 h (BV2 cells). Data are presented as mean ± S.E.M. N = 3.

Cell Line	Control	Hemo-Ionic
SH-SY5Y	100.0 ± 0.8	92.7 ± 1.8
BV2	100.0 ± 1.0	96.2 ± 1.6

## Data Availability

The original contributions presented in the study are included in the article, further inquiries can be directed to the corresponding authors.

## Abbreviations

The following abbreviations are used in this manuscript:

AAE	Ascorbic Acid Equivalents
ABTS	2,2′-Azino-bis(3-ethylbenzothiazoline-6-sulfonic acid)
ATCC	American Type Culture Collection
BHA	Butylated Hydroxyanisole
BHT	Butylated Hydroxytoluene
cDNA	Complementary DNA
CNS	Central Nervous System
CO_2_	Carbon Dioxide
COX-2	Cyclooxygenase-2
DPPH	2,2-Diphenyl-1-picrylhydrazyl
ELISA	Enzyme-Linked Immunosorbent Assay
FBS	Fetal Bovine Serum
GAPDH	Glyceraldehyde-3-phosphate Dehydrogenase
IL-1β	Interleukin-1 Beta
IL-6	Interleukin-6
iNOS	Inducible Nitric Oxide Synthase
LPS	Lipopolysaccharide
mRNA	Messenger Ribonucleic Acid
MTT	3-(4,5-dimethylthiazol-2-yl)-2,5-diphenyltetrazolium bromide
NF-κB	Nuclear Factor Kappa B
NO	Nitric Oxide
Nrf2	Nuclear Factor Erythroid 2–Related Factor 2
PBS	Phosphate-Buffered Saline
qRT-PCR	Quantitative Reverse Transcription Polymerase Chain Reaction
ROS	Reactive Oxygen Species
RPMI	Roswell Park Memorial Institute Medium
SDS	Sodium Dodecyl Sulfate
S.E.M.	Standard Error of the Mean
TNF-α	Tumor Necrosis Factor Alpha
TRP	Total Reducing Power
